# HSP90 Enhances Mitophagy to Improve the Resistance of Car-Diomyocytes to Heat Stress in Wenchang Chickens

**DOI:** 10.3390/ijms252111695

**Published:** 2024-10-30

**Authors:** Jiachen Shi, Zeping Ji, Xu Yao, Yujie Yao, Chengyun Li, Qijun Liang, Xiaohui Zhang

**Affiliations:** 1 Key Laboratory of Tropical Animal Breeding and Epidemic Disease Research of Hainan Province, School of Tropical Agriculture and Forestry, Hainan University, Haikou 570100, China; jiachenshi@hainanu.edu.cn (J.S.); old-chickin@hainanu.edu.cn (Z.J.); 21210905000016@hainanu.edu.cn (X.Y.); yujieyao@hainanu.edu.cn (Y.Y.); lcy0130@hainanu.edu.cn (C.L.); 21220951330003@hainanu.edu.cn (Q.L.); 2 School of Life and Health Sciences, Hainan University, Haikou 570228, China

**Keywords:** HSP90, mitophagy, heat stress damage, cardiomyocytes, Wenchang chicken

## Abstract

Heat shock protein 90 (HSP90) is recognized for its protective effects against heat stress damage; however, the specific functions and underlying molecular mechanisms of HSP90 in heat-stressed cardiomyocytes remain largely unexplored, particularly in tropical species. In our study, Wenchang chickens (WCCs) were classified into two groups: the heat stress survival (HSS) group and the heat stress death (HSD) group, based on their survival following exposure to heat stress. Heat stress resulted in significant cardiomyocyte damage, mitochondrial dysfunction, and apoptosis in the HSD group, while the damage was less pronounced in the HSS group. We further validated these findings in primary cardiomyocytes derived from Wenchang chickens (PCWs). Additionally, heat stress was found to upregulate Pink1/Parkin-mediated mitophagy, which was accompanied by an increase in HSP90 expression in both cardiomyocytes and PCWs. Our results demonstrated that HSP90 overexpression enhances PINK1/Parkin-mediated mitophagy, ultimately inhibiting apoptosis and oxidative stress in heat-stressed PCWs. However, the application of Geldanamycin (GA) reversed these effects. Notably, we discovered that HSP90 interacts with Beclin-1 through mitochondrial translocation and directly regulates mitophagy levels in PCWs. In summary, we have elucidated a novel role for HSP90 and mitophagy in regulating heat stress-induced acute cardiomyocyte injury.

## 1. Introduction

Heat stress is an increasingly significant public health issue globally [1]. HS has negative effects on the health of humans and animals, which can even cause sudden death [2]. As climate change and extreme heat events become more pronounced, the severity of these adverse effects is likely to escalate. For instance, this summer, temperatures in the Indian region peaked at 52.3 °C, resulting in over 211 heat-stress-induced fatalities. Chickens are recognized as an optimal animal model for investigating heat stress because of their sensitivity to heat stress and narrow thermal comfort zone [3,4]. The primary cause of sudden death in heat-stressed chickens is acute heart failure, which is closely associated with cardiac structural damage during heat stress [5]. Previous studies have demonstrated that heat stress induces various pathological changes in the hearts and myocardial cells of poultry, including swelling, degeneration, necrosis, and rupture of myocardial fibers [6,7].

Unregulated oxidative stress often leads to severe cellular damage and unwanted cell death, ultimately resulting in complete organ failure. Oxidative stress occurs when the intracellular levels of reactive oxygen species (ROS) in cardiomyocytes exceed the capacity of antioxidant defenses, with mitochondria serving as the principal source of these ROS [8]. Mitochondrial dysfunction poses a fundamental challenge in heart failure, and mitophagy has been recognized as a critical mechanism for maintaining mitochondrial quality control in this context [9]. Mitophagy is a biological process through which damaged or dysfunctional mitochondria are selectively cleared, thereby preserving mitochondrial homeostasis by regulating their quantity [10]. The cardioprotective effect induced by mitophagy has been demonstrated to be more enduring than that of general autophagy, with Beclin-1 and PINK1 identified as key contributors to this protective mechanism [11]. Partial restoration of mitochondrial phagocytosis has been shown to significantly improve cardiac function in an acute heart failure model [12], although excessive mitochondrial phagocytosis can also lead to cardiomyocyte damage and animal mortality [13]. For example, acute heat stress disrupts mitochondrial homeostasis in animals, prompting the occurrence of mitophagy [14]. Additionally, another study revealed that acute heat stress injury in duck ovarian granulosa cells was mitigated by the regulation of mitophagy [15]. Thus, the role of mitophagy in cardiomyocytes under heat stress conditions in chickens requires further investigation.

Heat shock proteins (HSPs) are crucial for safeguarding various cellular processes and preserving the integrity of cells under heat stress, thus improving the organism’s overall stress tolerance. Among these proteins, HSP90, an ATP-dependent molecular chaperone, is involved in the maturation of approximately 10% of the proteome [16]. Additionally, HSP90 also plays a crucial role in protein folding, maturation, and the preservation of cellular homeostasis [17,18]. Studies have demonstrated that the upregulation of HSP90 in chicken myocardial cells, triggered by aspirin during the initial phase of heat stress, results in a significant reduction in both the severity of heat-stress-induced damage and the apoptosis rate of cardiomyocytes [6,19]. This process involves the activation of the Akt-Bcl-2 and Raf-1-ERK signaling pathways by HSP90, which inhibit mitochondrial calcium overload, thereby preserving the function and homeostasis of heat-stressed cardiomyocytes [5,20]. These findings suggest that HSP90 is instrumental in maintaining mitochondrial function and reducing apoptosis. However, the specific mechanisms by which HSP90 sustains mitochondrial homeostasis in chickens have yet to be fully elucidated.

Wenchang chicken (WCC), a representative tropical poultry species, is recognized for its significant heat resistance, resulting in fewer heat-stressed fatalities compared to other poultry species [21]. The notable heat resistance of WCC may serve as a general reference for developing strategies to mitigate heat stress-related mortality in humans and other animals. Therefore, investigating the mechanisms underlying WCCs’ resistance to heat stress is essential. This study examines the pathological damage to Wenchang chicken cardiomyocytes under heat stress, with a particular focus on the role and mechanism of HSP90 in counteracting heat stress-induced damage. We establish a heat stress model in WCCs and analyze the pathological features between surviving and nonsurviving groups. These findings are further validated using primary cardiomyocytes from Wenchang chickens (PCWs). Additionally, we investigate the involvement of HSP90 and mitophagy in the molecular mechanisms that underlie heat stress in cardiomyocytes. These results provide valuable insights into the mechanisms of heat stress resistance in tropical poultry, particularly concerning cardiac health, and offer critical information for developing intervention strategies to enhance heat stress resistance in humans and various other animals.

## 2. Results

### 2.1. Establishment of Heat-Stressed Cardiomyocyte Models In Vitro and In Vivo in WCCs

**Figure 1:**
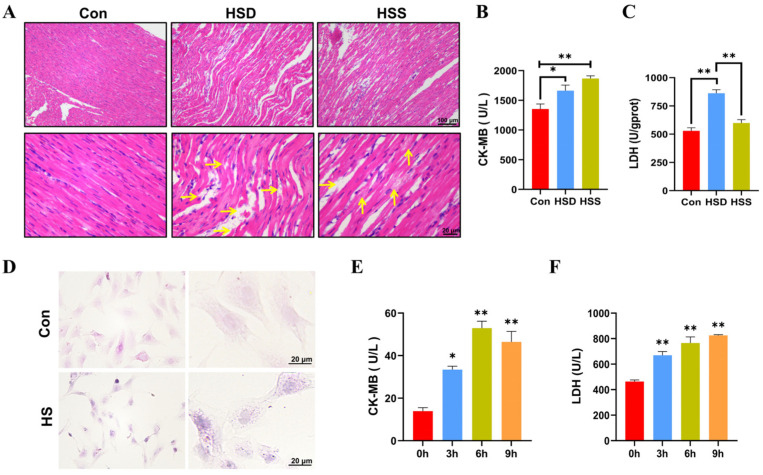
Establishment of heat-stressed cardiomyocyte models in vitro and in vivo in WCCs. (**A**) Myocardial tissues in WCCs were stained by H&E staining. Scale bars: 2 μm (**top**) and 500 nm (**bottom**), n = 3. Vertical yellow arrow: swelling of cardiac fibers, horizontal yellow arrow: myocardial fiber rupture. (**B**) The levels of CK-MB in the serum of heat-stressed WCCs were detected. Data are presented as the mean ± SEM. * *p* < 0.05, ** *p* < 0.01, n = 3. (**C**) The levels of LDH in the myocardial tissue of heat-stressed WCCs were detected. Data are presented as the mean ± SEM. ** *p* < 0.01, n = 3. (**D**) Heat-stressed PCWs for 3 h (HS) were stained by H&E (scale bar: 20 μm). In each group, the image on the right is a partial enlargement of the image on the left. (**E**) The levels of CK-MB in the supernatant of heat-stressed PCWs cultures were measured. * *p* < 0.05, ** *p* < 0.01, n = 3. (**F**) The levels of LDH in the supernatant of heat-stressed PCWs cultures were measured (n = 3). The differences in the data of cells heat-stressed for different times vs. those heat-stressed for 0 h are indicated by * *p* < 0.05 and ** *p* < 0.01, n = 3.

To evaluate the degree of myocardial injury, hematoxylin–eosin (H&E) stained sections were performed. As noticed, the myocardium in the control (Con) group presented the characteristics of normal structure with regularly arranged and densely packed myofibrils. In comparison to the Con group, heat stress induced significant myocardial injury, as evidenced by the swelling of myocardial fibers, widening of the interstitial spaces, karyopyknosis, and rupture of myocardial fibers in the heat stress death (HSD) group. In contrast, the heat stress survival (HSS) group displayed only mild swelling, granular degeneration, and occasional lipid droplet formation (Figure 1A). We also measured serum markers of myocardial injury, specifically lactate dehydrogenase (LDH) and creatine kinase-MB (CK-MB), and found that the levels of both LDH and CK-MB were significantly elevated in the HSD group compared to the Con group. Additionally, CK-MB levels were significantly increased in the HSS group, while LDH levels exhibited a slight elevation (Figure 1B,C). These findings suggest that heat stress inflicts structural as well as functional impairments on the myocardium of WCCs. It is noteworthy that the degree of injury in the HSS group was less pronounced compared to that in the HSD group. To further investigate the effects of heat stress on cardiomyocytes, we isolated PCWs. Following a 3-h exposure to heat stress, we documented structural modifications in PCWs, such as cytoplasmic reduction, granular degeneration, and nuclear retraction (Figure 1D). In comparison to 0 h, the levels of LDH and CK-MB in the cell culture supernatant significantly increased after 3 to 9 h of heat stress, indicating that the PCWs suffered serious damage under heat stress (Figure 1E,F). These results indicate that cardiomyocyte injury models were successfully established in vivo and in vitro in WCCs.

### 2.2. Heat Stress Induces Mitochondrial Dysfunction and Oxidative Stress in Cardiomyocytes of WCCs

**Figure 2:**
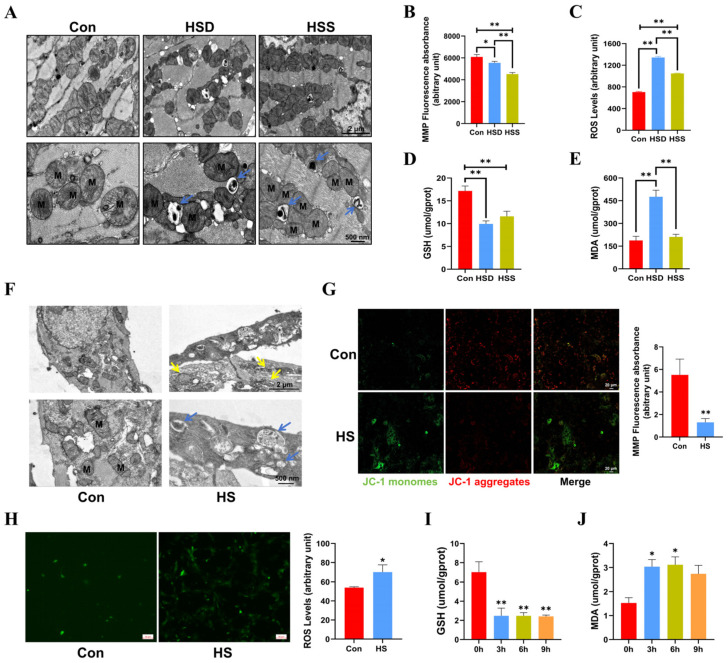
Heat stress induces mitochondrial dysfunction in cardiomyocytes and PCWs, leading to oxidative stress. (**A**) TEM images showing mitochondrial morphology and autophagosomes in heat-stressed cardiomyocytes. M indicates mitochondria; blue arrows point to mitochondrial autophagosomes. Scale bars: 2 μm (**top**) and 500 nm (**bottom**). (**B**) The mitochondrial membrane potential (MMP) in the myocardial tissue of heat-stressed WCCs were detected; data are presented as the mean ± SEM. * *p* < 0.05, ** *p* < 0.01, n = 3. The levels of ROS (**C**), GSH (**D**), and MDA (**E**) in the myocardial tissue of heat-stressed WCCs were detected. Data are presented as the mean ± SEM. ** *p* < 0.01, n = 3. (**F**) TEM images showing the presence of abnormally shaped mitochondria and autophagosomes in the Con group and heat-stressed PCWs for 3 h (HS). Blue arrows point to autophagosomes, and yellow arrows point to mitochondria with disrupted cristae. Scale bars: 2 μm (**top**) and 500 nm (**bottom**), n = 3. (**G**) PCWs were treated with heat stress for 3 h and then treated with a JC-1 probe and observed under a fluorescence microscope. The representative images for each condition are shown. Scale bars: 20 μm. Red fluorescence represents JC-1 aggregates, and green fluorescence represents JC-1 monomers, ** *p* < 0.01, n = 3. (**H**) The ROS levels of PCWs were determined after exposure to heat stress for 3 h. * *p* < 0.05, n = 3. The GSH (**I**) and MDA (**J**) levels of PCWs were determined after exposure to heat stress for different times. The differences in the data of cells heat-stressed for different times vs. those heat-stressed for 0 h are indicated by * *p* < 0.05, ** *p* < 0.01, n = 3.

Cardiomyocytes possess one of the highest mitochondrial contents, with approximately 30% of their volume comprising mitochondria [22]. To assess the impact of heat stress on mitochondrial morphology in cardiomyocytes, we employed transmission electron microscopy (TEM). The results revealed that some mitochondria exhibited disordered cristae and abnormal structures in the HSS group. Additionally, mitochondrial cristae disorders and vacuolization were noted in the HSD group, with mitophagosomes identified in both the HSS and HSD groups (Figure 2A). Mitochondrial function is closely linked to mitochondrial morphology, and the mitochondrial membrane potential (MMP) and reactive oxygen species (ROS) are critical indicators for evaluating the structural and functional integrity of mitochondria [23]. Consequently, we measured MMP and ROS levels in cardiomyocytes from WCCs. The results indicated a substantial reduction in MMP, coupled with a remarkable elevation in ROS levels within cardiac tissues exposed to heat stress, suggesting that heat stress elicits mitochondrial dysfunction in cardiac tissue (Figure 2B,C). We subsequently examined the levels of malondialdehyde (MDA) and glutathione (GSH) to assess whether heat stress induces oxidative damage in the cardiac tissues. In comparison to the Con group, the levels of GSH in the cardiac tissues of the HSS and HSD groups exhibited a significant decrease, while the MDA levels in the HSD group showed a significant increase (Figure 2D,E). Furthermore, in light of the evident damage in PCWs induced by 3 h of heat stress, we first examined mitochondrial morphology. The results revealed abnormal mitochondrial morphology and disrupted cristae. Notably, we also identified autophagosomes containing undegraded organelles, including mitochondria (Figure 2F). Additionally, heat stress significantly reduced MMP levels (Figure 2G) and increased ROS levels in PCWs (Figure 2H). In comparison to the 0-h time point, GSH levels in PCWs significantly decreased at 3, 6, and 9 h of heat stress. Conversely, MDA levels significantly increased at 3 and 6 h, exhibiting an upward trend at 9 h of heat stress (Figure 2I–J). These findings suggest that heat stress can induce oxidative stress injury and cause mitochondrial structural damage in the cardiomyocytes of WCCs. 

### 2.3. The Effects of Heat Stress on Apoptosis in Cardiomyocytes of WCCs

**Figure 3:**
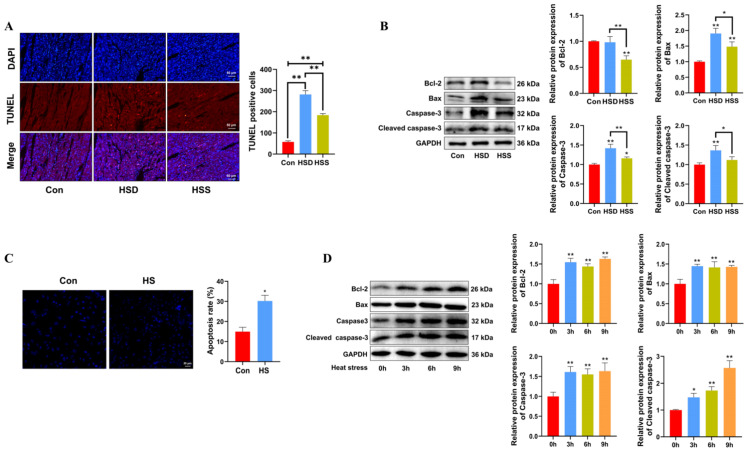
Heat stress induces apoptosis in cardiomyocytes and PCWs. (**A**) TUNEL staining was performed in the myocardial tissue of WCCs following treatment with heat stress. Apoptosis was determined by TUNEL staining (red) and the nuclei were stained with DAPI (blue). TUNEL positive ratio is defined as the number of TUNEL positive cells divided by the total number of cells. Values are presented as the mean ± SEM of three individual experiments, Scale bars: 50 μm, ** *p* < 0.01, n = 3. (**B**) Measurement of protein expression of apoptosis-related proteins Bcl-2, Bax, Caspase-3, and cleaved caspase-3 in myocardial tissues of WCCs treated with heat stress by Western blot analysis, normalized to GAPDH. Measurement data are expressed as mean ± SEM. * *p* < 0.05, ** *p* < 0.01, n = 3. (**C**) Hoechst 33342 staining was performed in heat-stressed PCWs for 3 h, scale bars: 50 μm, * *p* < 0.05, n = 3. (**D**) After exposure to heat stress for different times, Bcl-2, Bax, Caspase-3, and Cleaved caspase-3 in whole cells were detected via Western blot analysis with the indicated antibodies. Representative bands are shown. The differences in the data of cells heat-stressed for different times vs. those heat-stressed for 0 h are indicated by * *p* < 0.05 and ** *p* < 0.01.

Apoptosis is a critical process in heat stress-induced cardiac damage. To assess the impact of heat stress on cardiomyocyte apoptosis, TUNEL staining and Western blot were conducted on WCCs’ cardiac tissues. The TUNEL assay revealed an increase in the number of apoptotic TUNEL+ cells in both the HSD and HSS groups, while the HSS group had significantly fewer TUNEL+ cells compared to the HSD group (Figure 3A). Relative to the Con group, the HSS group demonstrated a reduction in the expression of the antiapoptotic protein Bcl-2 and an increase in the expression of the proapoptotic protein Bax, indicating that heat stress activates apoptotic pathways. Furthermore, the notable increase in levels of apoptosis-related proteins, specifically cleaved caspase-3 and Caspase-3, corroborated these findings (Figure 3B). Consistent with in vivo results, PCWs subjected to 3 h of heat stress demonstrated significantly elevated levels of apoptosis (Figure 3C), accompanied by increased expression of cleaved caspase-3, Caspase-3, and Bax (Figure 3D). Collectively, these findings indicate that heat stress induces apoptosis in the cardiomyocytes of WCCs.

### 2.4. Heat Stress Induces Mitophagy and Increases HSP90 Expression in Cardiomyocytes of WCCs and PCWs

**Figure 4:**
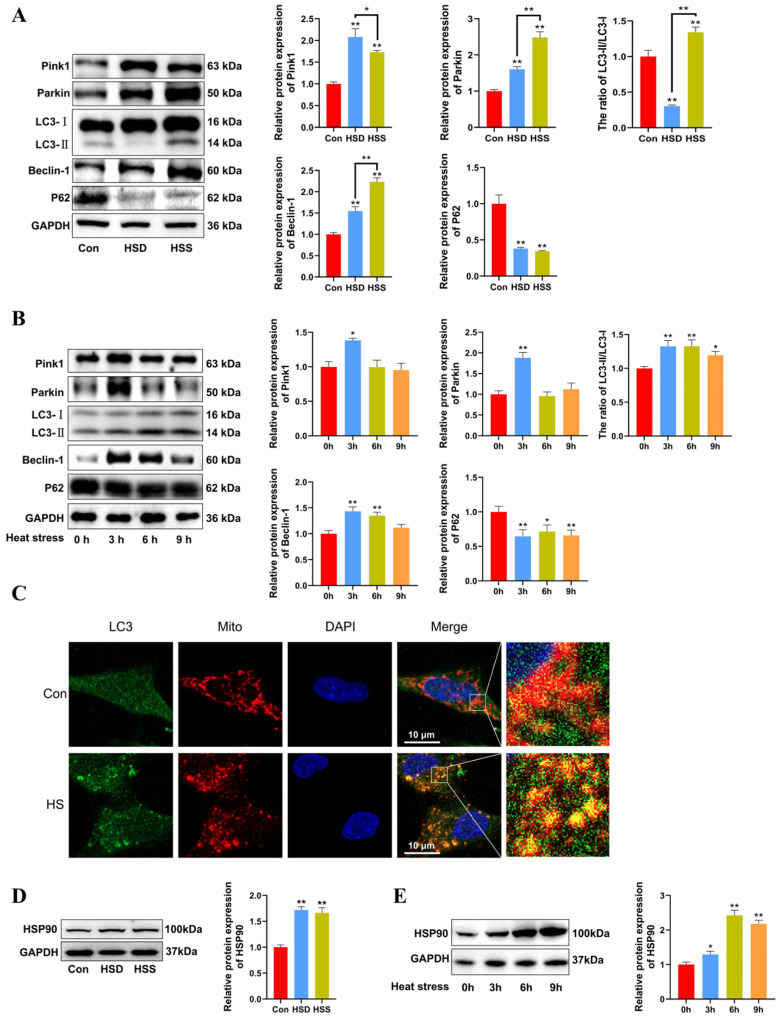
Heat stress induces mitophagy and high HSP90 expression in cardiomyocytes and PCWs. (**A**) Measurement of protein expression of mitophagy-related proteins Pink1, Parkin, LC3, Beclin-1, and P62 in myocardial tissues of WCCs treated with heat stress by Western blot analysis, normalized to GAPDH. Measurement data are expressed as mean ± SEM. * *p* < 0.05, ** *p* < 0.01, n = 3. (**B**) After exposure to heat stress for different times, Pink1, Parkin, LC3, Beclin-1, and P62 in whole cells were detected via Western blot analysis with the indicated antibodies. Representative bands are shown. The differences in the data of cells heat-stressed for different times vs. those heat-stressed for 0 h are indicated by * *p* < 0.05 and ** *p* < 0.01. (**C**) Immunofluorescence staining of LC3 dots (LC3, green) and Mito Tracker (Mito, red) in heat-stressed PCWs for 3 h. Scale bars: 10 μm, n = 3. (**D**) Measurement of protein expression of HSP90 in myocardial tissues of WCCs treated with heat stress by Western blot analysis, normalized to GAPDH. Measurement data are expressed as mean ± SEM. ** *p* < 0.01, n = 3. (**E**) After exposure to heat stress for different times, HSP90 in whole cells were detected via Western blot analysis with the indicated antibodies. Representative bands are shown. The differences in the data of cells heat-stressed for different times vs. those heat-stressed for 0 h are indicated by * *p* < 0.05 and ** *p* < 0.01.

We previously observed mitophagosomes in heat-stressed cardiomyocytes and PCWs. We hypothesize that mitophagy may play a protective role in heat-stressed cardiomyocytes. To investigate this, we examined the levels of mitophagy-related proteins using Western blot in heat-stressed cardiac tissues. The results indicated that the LC3-II/LC3-I ratio was significantly decreased in the HSD group but significantly increased in the HSS group. Furthermore, compared to the Con group, the levels of Beclin-1, Pink1, and Parkin were significantly elevated, while P62 levels were markedly reduced in the cardiac tissues of both the HSD and HSS groups (Figure 4A). Compared with 0 h, there were significant increases in the LC3-II/LC3-I ratio in the heat-stressed PCWs at 3, 6, and 9 h, accompanied by a notable decrease in P62 levels. Additionally, the expression of Beclin-1, Pink1, and Parkin significantly increased after 3 h of heat stress (Figure 4B). The fluorescent signals indicating mitochondrial colocalization with LC3 in PCWs significantly increased after 3 h of heat stress (Figure 4C). These findings suggest that heat stress initiates mitophagy in cardiomyocytes of WCCs. HSP90 serves as a major hub within the molecular chaperone network and regulates mitochondrial autophagy by stabilizing client proteins [24]. To evaluate HSP90 expression during heat stress, we assessed the protein levels of HSP90 using Western blott. The results demonstrated that HSP90 expression levels were significantly upregulated in the cardiac tissues of both the HSS and HSD groups (Figure 4D). Furthermore, we analyzed HSP90 expression levels in heat-stressed PCWs at various time points. Compared to the levels at 0 h, HSP90 expression was significantly elevated at 3, 6, and 9 h of heat stress (Figure 4E). These results indicate that heat stress induces mitophagy in cardiomyocytes and upregulates HSP90 expression in cardiomyocytes of WCCs.

### 2.5. Effect of HSP90 Overexpression on Heat-Stressed PCWs

**Figure 5:**
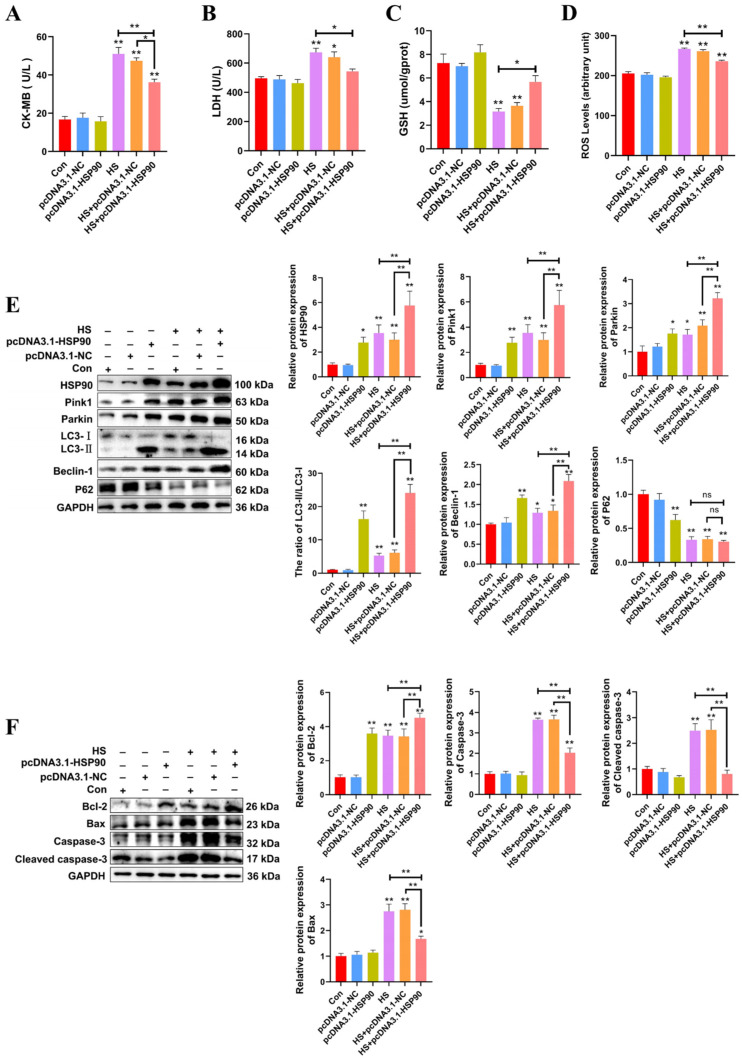
HSP90 overexpression reduces heat stress-induced PCWs damage and apoptosis, while enhancing mitophagy. (**A**,**B**) The levels of CK-MB and LDH in PCWs with or without HSP90 overexpression after heat stress. Measurement data are expressed as mean ± SEM. * *p* < 0.05, ** *p* < 0.01, n = 3. (**C**,**D**) The levels of ROS and GSH in PCWs with or without HSP90 overexpression after heat stress. Measurement data are expressed as mean ± SEM. * *p* < 0.05, ** *p* < 0.01, n = 3. (**E**) HSP90, Pink1, Parkin, LC3, Beclin-1, and P62 in PCWs with or without HSP90 overexpression after heat stress were detected via Western blot analysis with the indicated antibodies. Representative bands are shown. Data are expressed as the means ± SEM. * *p* < 0.05, ** *p* < 0.01, n = 3. (**F**) Bcl-2, Bax, Caspase-3, and cleaved caspase-3 in PCWs with or without HSP90 overexpression after heat stress were detected via Western blot analysis with the indicated antibodies. Representative bands are shown. Data are expressed as the means ± SEM. * *p* < 0.05, ** *p* < 0.01, n = 3.

The experiment demonstrates that PCWs subjected to 3 h of heat stress exhibit significant signs of thermal damage. Consequently, a duration of 3 h of heat stress was selected for the molecular experiments. To further investigate the influence of HSP90 on PCWs, we generated HSP90-overexpressing PCWs. H&E staining revealed that PCWs subjected to heat stress exhibited granule-like degeneration in the cytoplasm and karyopyknosis. In contrast, HSP90-overexpressing PCWs demonstrated reduced granular degeneration and alleviated nuclear condensation (Figure S1). When cultured under heat stress, HSP90-overexpressing PCWs exhibited significantly reduced levels of CK-MB, LDH, and ROS, while GSH levels significantly increased compared to PCWs under heat stress (Figure 5A–D). To further elucidate the impact of HSP90 on mitophagy-related proteins, we assessed the expression levels of these proteins in HSP90-overexpressing PCWs. Relative to the HS group, the LC3-II/LC3-I ratio and expression levels of Beclin-1, Pink1, and Parkin in the HS+pcDNA3.1-HSP90 group were significantly greater. There was no significant difference in the protein expression levels of p62 between the HS and HS+pcDNA3.1-HSP90 groups (Figure 5E). In contrast to PCWs under heat stress, HSP90-overexpressing PCWs displayed lower expression levels of cleaved caspase-3 and Bax, and elevated levels of Bcl-2 (Figure 5F). These findings indicate that HSP90 effectively mitigates heat stress-induced damage and apoptosis in PCWs while promoting mitophagy.

### 2.6. Effect of HSP90 Functional Inhibition on Heat-Stressed PCWs

**Figure 6:**
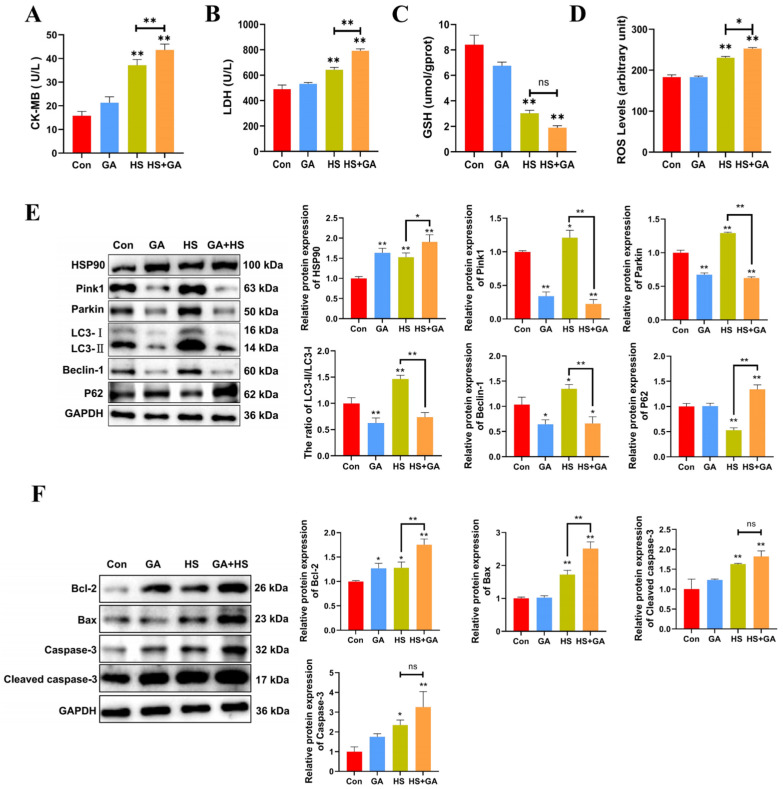
Inhibition of HSP90 function leads to increased apoptosis and damage, with downregulated mitophagy in heat-stressed PCWs. (**A**,**B**) The levels of CK-MB and LDH in PCWs treated with or without GA after heat stress. Measurement data are expressed as mean ± SEM. * *p* < 0.05, ** *p* < 0.01, n = 3. (**C**,**D**) The levels of ROS and GSH in PCWs treated with or without GA after heat stress. Measurement data are expressed as mean ± SEM. * *p* < 0.05, ** *p* < 0.01, n = 3. (**E**) HSP90, Pink1, Parkin, LC3, Beclin-1, and P62 in PCWs treated with or without GA after heat stress were detected via Western blot analysis with the indicated antibodies. Representative bands are shown. Data are expressed as the means ± SEM * *p* < 0.05, ** *p* < 0.01, n = 3. (**F**) Bcl-2, Bax, caspase-3, and cleaved-caspase-3 in PCWs treated with or without GA after heat stress were detected via Western blot analysis with the indicated antibodies. Representative bands are shown. Data are expressed as the means ± SEM. * *p* < 0.05, ** *p* < 0.01, n = 3.

We investigated the protective role of HSP90 under heat stress by utilizing geldanamycin (GA). H&E staining revealed that GA-treated heat-stressed PCWs exhibited a loss of normal morphology, severe cellular vacuolization, and the presence of apoptotic cells (Figure S2). Furthermore, in comparison to the HS group, GA-treated heat-stressed PCWs demonstrated significantly elevated levels of LDH, CK-MB, and ROS, alongside reduced GSH levels (Figure 6A–D). These findings suggest that the inhibition of HSP90 function exacerbates both cellular damage and oxidative stress. Additionally, GA-treated heat-stressed PCWs showed a significant decrease in the LC3-II/LC3-I ratio, as well as reduced levels of Beclin-1, Pink1, and Parkin, while exhibiting a marked increase in P62 expression (Figure 6E). We subsequently confirmed the proapoptotic effects of HSP90 inhibition through a significant increase in cleaved caspase-3 and Caspase-3 levels, as well as alterations in Bax and Bcl-2 expression (Figure 6F). Collectively, these results indicate that the inhibition of HSP90 function in heat-stressed PCWs leads to heightened apoptosis and cellular damage, accompanied by downregulated mitophagy.

### 2.7. Effect of Heat Stress on the Interaction Between HSP90 and Beclin-1

**Figure 7:**
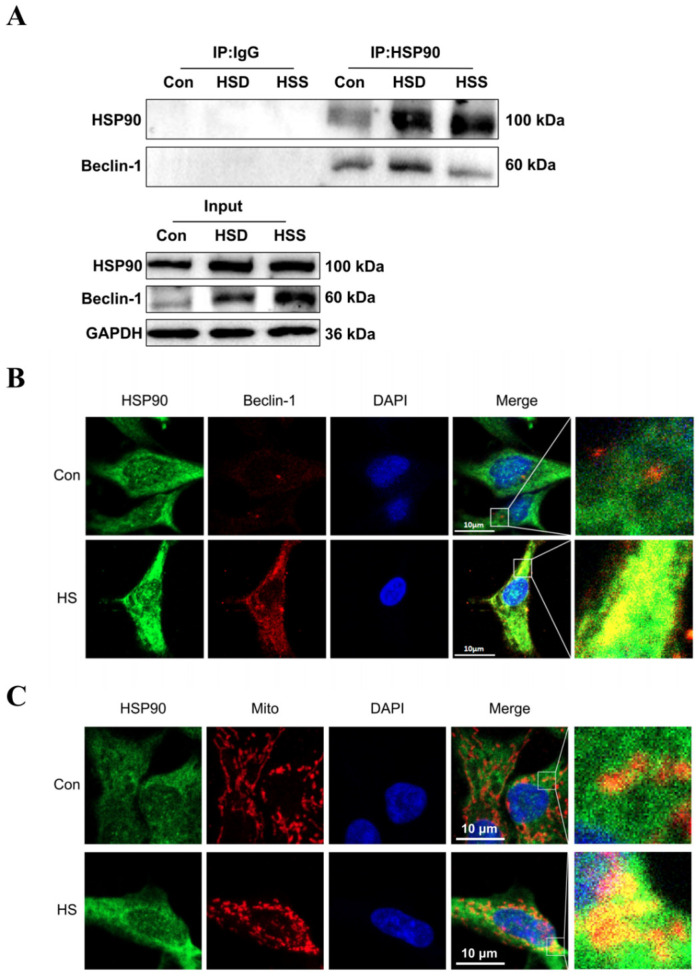
Effect of heat stress on the interaction between HSP90 and Beclin-1. (**A**) Coimmunoprecipitation (Co-IP) of HSP90 and Beclin-1 in the myocardial tissue of WCCs following treatment with heat stress. (**B**) Immunofluorescence staining of HSP90 (HSP90, green) and Beclin-1 (Beclin-1, red) in PCWs heat-stressed for 3 h, scale bars: 10 μm, n = 3. (**C**) Immunofluorescence staining of HSP90 (HSP90, green) and Mito Tracker (Mito, red) in PCWs heat-stressed for 3 h, scale bars: 10 μm, n = 3.

Previous studies have demonstrated that Beclin-1 serves as a common marker of mitophagy, while HSP90 can form a complex with Beclin-1 through its conserved domain, thereby maintaining Beclin-1 stability and regulating autophagy [25]. To investigate whether HSP90 directly interacts with Beclin-1 in heat-stressed cardiomyocytes of WCCs, we conducted coimmunoprecipitation (Co-IP) experiments. The results revealed specific interactions between HSP90 and Beclin-1 in the cardiac tissues of WCC from the Con, HSS, and HSD groups (Figure 7A). Furthermore, in heat-stressed PCWs, increased signals for HSP90 and Beclin-1 were strongly colocalized in the cytoplasm (Figure 7B). Additionally, elevated levels of HSP90 and intensified colocalization signals of HSP90 with mitochondria were observed in heat-stressed PCWs, suggesting that mitochondrial translocation of HSP90 occurs under heat stress conditions (Figure 7C). These findings indicate that during heat stress, HSP90 may regulate mitophagy by enhancing its interaction with Beclin-1.

## 3. Discussion

Chickens possess a limited capacity for body temperature regulation and are particularly vulnerable to sudden death due to heat stress [26]. Heat-stressed broiler chickens demonstrate myocardial fiber breakage, mitosis, and degeneration, accompanied by significant increases in myocardial-injury-related enzymes, indicating substantial cardiac damage resulting from heat stress [27]. Our study revealed that the HSD group exhibited pathological changes characteristic of heart failure, including myocardial fiber breakage and cellular swelling. Notably, LDH levels in the HSS group were significantly lower than those in the HSD group. Oxidative stress was induced in chicken cells due to heat stress, with key indicators being MDA and GSH [28]. The GSH levels in heat-stressed broiler chickens significantly decreased, while MDA levels significantly increased [29]. In this study, the MDA and GSH levels in the HSD group indicated the presence of oxidative stress in the cardiac tissues of WCCs. Conversely, no significant difference in MDA content was observed between the HSS and Con groups, suggesting a lower level of oxidative stress in the HSS group. Mitochondria, being the most vulnerable organelles in cardiomyocytes, frequently undergo morphological changes during heat stress in animals [20,30]. We observed that the HSS group exhibited mitochondrial damage compared to the control group, while the HSD group displayed even more severe mitochondrial damage. Interestingly, we identified mitochondrial autophagosomes in both the HSS and HSD groups, indicating a connection between heat stressinduced mitochondrial damage and mitophagy.

Mitochondria constitute approximately 30% of the volume of cardiomyocytes and are responsible for supplying over 90% of the ATP required for myocardial contraction, thereby establishing a close relationship between myocardial cell functions and mitochondrial activity [31]. These organelles are recognized as critical determinants of cellular survival or death, making the maintenance of stable mitochondrial function essential for overall survival [32]. This study shows that heatstress induced a decrease in the activity of the mitochondrial complex and ATPase in the chicken spleen [33]. In addition, heat stress induced specific internal environmental states in rat cardiomyocyte mitochondria, including Ca^2+^ overload, oxidative stress, and altered mitochondrial membrane permeability transition [34]. These changes result in mitochondrial damage in rat myocardial cells, which subsequently leads to cardiac dysfunction and failure. Damaged mitochondria can release signaling molecules such as cytochrome c, thereby activating autophagy-related signaling pathways [35,36]. Autophagy is initiated by a range of physiological and pathological processes within the cell, including organelle damage and stress. During autophagy, specific receptor proteins, such as PINK1 and Park2, recognize damaged mitochondria, encapsulate them, and transport them to lysosomes for degradation. This process is essential for maintaining mitochondrial quality and function [37].

Mitophagy, a form of selective autophagy within macroautophagy, is classified into ubiquitin-dependent and ubiquitin-independent mitophagy [38]. Ubiquitin-dependent mitophagy, mediated by PINK1 and Parkin, plays a critical role in the degradation of damaged mitochondria in mammals [39]. Upon activation, PINK1 exhibits kinase activity, which promotes the phosphorylation and translocation of Parkin to the outer mitochondrial membrane, thereby activating Parkin to ubiquitinate outer membrane proteins and form polyubiquitin chains. Subsequently, PINK1 further phosphorylates these chains, and the phosphorylated polyubiquitin chains are recognized by autophagy adaptors, which ultimately bind to LC3 to form autophagosomes, completing the autophagic degradation process. The initiation of mitophagy and autophagy is generally associated with increased mitochondrial Parkin levels [40]. The induction of mitophagy relies on the production of ROS, whereas the inhibition of autophagy or mitophagy leads to increased ROS production [41]. Our results indicate that heat stress severely disrupts the structural and functional integrity of mitochondria in the cardiomyocytes of WCCs, significantly elevating ROS levels and inducing mitophagy in these cells. Additionally, this study found higher levels of mitophagy in the HSS group compared to the HSD group, suggesting that mitophagy may contribute to the resistance of WCCs to heat stress. However, due to the faster mortality observed in the HSD group, these chickens were unable to mitigate heat stress through mitophagy in a timely manner.

If damaged mitochondria within cells are not effectively removed through mitophagy, this can lead to caspase-mediated apoptosis [42]. Excessive cell apoptosis in cardiac tissue has been linked to heart failure [43,44]. Research indicates that heat stress induces apoptosis in the tissues and organs of white-feathered broilers, rats, and cattle [45,46]. This study confirmed the initiation and occurrence of apoptosis in both in vivo and in vitro cardiomyocyte models under heat stress. These findings suggest that apoptosis may play a critical role in heat-stress induced sudden death. Generally, HSP90 exhibits a distinct antiapoptotic function and can regulate the expression of antiapoptotic proteins [47]. After suppressing the chaperone function of myocardial HSP90, we observed that heat stress-induced damage and apoptosis were further exacerbated. Conversely, the overexpression of HSP90 demonstrated significant protective effects against apoptosis. However, myocardial cells treated with heat stress plus GA displayed high levels of Bcl-2 but did not exhibit antiapoptotic effects. This may be due to the presence of Bcl-2 phosphorylation and Bcl-2/Bcl-X dimers, which weaken the antiapoptotic capacity of Bcl-2 [48,49].

HSPs are essential for preventing cellular damage and enhancing resistance to heat stress [50]. Increased expression of HSP90 in the PCWs exposed to heat stress has been shown to provide significant protective effects [51]. HSP90 is a molecular chaperone in the form of dimers: the conformation of the N-terminal structural domain changes upon binding to ATP, the intermediate structural domain forms a complex with the chaperone molecules and binds to the client proteins, and the N-terminal structural domains at both ends of the dimer form a closed state. Upon hydrolysis by the ATPase, the conformation of cytoplasmic HSP90 is altered and reverts to the original open state, the co-chaperone molecules are disassociated from the intermediate structural domain, and the correctly folded client proteins are released [52]. Among them, HSP90 is closely related to oxidative stress and mitochondrial dysfunction [53]. HSP90 is vital for maintaining the vitality of boar sperm and preserving the MMP against heat stress damage [54]. Consistent with previous studies, the upregulation of HSP90 in the cardiomyocytes of WCCs following heat stress further supports the potential of HSP90 as a biomarker for heat stress. As a chaperone, HSP90 can form complexes with its cochaperones, such as HSP70, to perform protective functions within cells [55]. This study indicates that the protein levels of HSP90 in the HSD group are slightly elevated compared to those in the HSS group. This increase may be attributed to the heightened demand for HSPs to maintain cellular protein homeostasis under heat stress, resulting in consumptive degradation [19,56]. Furthermore, HSP90 is upregulated in an in vitro heat stress model of PCWs, suggesting that HSP90 may play a more enduring role in WCCs during heat stress.

HSP90-mediated autophagic protective pathways provide an effective self-protection and repair mechanism for cells. Heat stress-induced HSP90 expression maintains bovine kidney cell survival by inhibiting cell apoptosis and increasing cell autophagy [57]. Additionally, HSP90 can activate the HIF-1α-BNIP3/BNIP3L pathway to stimulate autophagy-mediated survival, protecting the body from heat stress damage [58]. Research indicates that HSP90 can activate AKT, thereby directly phosphorylating mTOR [59]. When the activity of mTOR is decreased, the inhibitory effect on the autophagy-related protein ULK1 will be reduced, thus inducing the occurrence of autophagy [60]. The inhibition of HSP90 promotes the degradation of autophagy-related proteins ATG7, Beclin-1, and ULK1 in cardiomyocytes, thus suppressing the autophagic process [61]. HSP90 interacts with CDC37, promotes Ulk1-mediated phosphorylation and the release of ATG13, and enhances autophagy to maintain cell stability [62]. Following the clearance of damaged mitochondria, mitophagy serves to inhibit myocardial cell apoptosis and mitigate the progression of heart failure [63]. This study demonstrated that PCWs overexpressing HSP90 exhibited increased levels of mitochondrial autophagy and antioxidant activity, which were reversed upon the inhibition of HSP90 function. Beclin-1 facilitates the formation of autophagosomes from damaged mitochondria and plays a pivotal role in the mitophagy process [64]. Beclin-1 is among the numerous client proteins of HSP90; after inhibiting HSP90 in A549 cells with Gedunin, the interaction among HSP90, Beclin-1, and Bcl-2 was diminished, leading to the induction of apoptosis [65]. In mice acutely exposed to arsenic, the upregulation of HSP90 was found to be essential for stabilizing the client protein Beclin-1 during autophagy [66]. Our study revealed that the enhanced interaction between HSP90 and Beclin-1 is critical for regulating Pink1/Parkin-mediated mitophagy in cardiomyocytes under acute heat stress. Conversely, the inhibition of the chaperone HSP90 resulted in a decrease in mitochondrial phagocytosis within cardiomyocytes.

## 4. Materials and Methods

### 4.1. Animal Handling and Sample Collection

A total of 40 male Wenchang chickens, aged 70 days and weighing between 700 and 750 g, were purchased from Longquan Wenchang Chicken Company in Hainan, China. All chickens were reared under standard laboratory conditions, which included a temperature of 22 ± 2 °C, relative humidity of 50 ± 10%, and a 12-h light/dark cycle, with ad libitum access to food and water. After a 1-week acclimation period, 34 healthy chickens with comparable body weights were divided into two groups: the heat stress group (n = 24) and the Con group (n = 10). The heat stress group was exposed to 42 ± 1 °C with a relative humidity of 65% for 5 consecutive hours, with free access to food and water. The Con group was maintained under normal conditions for the same duration. Of the chickens, 9 that died during the heat stress exposure were assigned to the HSD group, and 15 that survived at the end of the exposure were assigned to the HSS group. Blood and cardiac tissues of chickens in the HSD group were rapidly collected within five minutes of death. Chickens in the HSS group were humanely slaughtered at the end of the experimental period. Subsequently, blood and cardiac tissues were rapidly collected for future experiments. The experimental protocol was guided and approved by the Animal Ethics Review Committee of Hainan University.

### 4.2. Isolation and Culture of PCWs

Embryos aged 12 days were sourced from Longquan Wenchang Chicken Company. The hearts were sectioned into tissue blocks measuring 1–2 mm^3^ and subsequently washed with precooled PBS. The residual ventricular tissue was subjected to repeated digestion using 0.1 mg/mL collagenase type I (Solarbio Life Sciences, Beijing, China) for 30 min at 37 °C until complete digestion of the tissue mass was achieved. Following centrifugation at 1000 r/min for 6 min, the resultant cardiomyocytes were collected and cultured in cell culture dishes at 37 °C for 2 h. The supernatant, which contained the ventricular cardiomyocytes, was carefully aspirated. The remaining suspension was then inoculated into high-glucose Dulbecco’s Modified Eagle’s Medium (DMEM, Cytiva, Marlborough, MA, USA), supplemented with 20% (*v*/*v*) fetal bovine serum (FBS, Biological Industries, Haemek, Israel) and 1% (*v*/*v*) penicillin–streptomycin (Solarbio Life Sciences, Beijing, China), and incubated for 48 h at 37 °C in a carbon dioxide incubator.

### 4.3. Activities of LDH and CK-MB in the Cardiac Tissues and PCWs

LDH levels in chicken heart homogenates and cell culture supernatant were measured using a microplate reader (Jiancheng Bioengineering, Nanjing, China) in accordance with the manufacturer’s guidelines. The concentration of CK-MB in the serum of WCCs and cell culture supernatants was determined using the immunosuppression method (Jiancheng Bioengineering, Nanjing, China).

### 4.4. Histopathologic Assay in the Cardiac Tissues and PCWs

Cardiac tissues were fixed in 10% formalin and embedded in paraffin, with sections cut to a thickness of 4.5 μm and stained using H&E staining. For cytopathological observation, cells were cultivated on climbing flakes and treated according to the experimental design. Initially, cells on the climbing flakes were observed using a light microscope, after which they were fixed with a 4% polyformaldehyde solution for H&E staining. Images of the stained sections were captured using a light microscope (Nikon Eclipse TE2000-U, Tokyo, Japan).

### 4.5. TEM in the Cardiac Tissues and PCWs

Cardiac tissues were cut into homogeneous cubes measuring 1.0 × 1.0 × 1.0 mm. These tissue samples were immediately immersed in a 2.5% glutaraldehyde phosphate-buffered saline solution and fixed for 12 h. Following three washes with PBS, the chicken heart blocks were subsequently fixed in a 1% osmium tetroxide solution for 1 to 2 h. The samples were then dehydrated using various concentrations of ethanol before being embedded in epoxy resin. An ultrathin microtome was employed to section the samples to a thickness of 70–90 nm. The sections were stained with lead citrate staining solution and dioxygen acetate axis staining solution, and subsequently examined using a transmission electron microscope (JEM-1200EX, Tokyo, Japan). For the TEM analysis of PCWs, the fixed cells were sent to Beijing Zhongkebaice Technology Service Co., Ltd., Beijing, China.

### 4.6. Measurement of MDA, GSH and ROS in the Cardiac Tissues

The cardiac tissue homogenate was centrifuged at 3000 r/min for 15 min to obtain the supernatant, which was subsequently used to measure the levels of MDA and GSH. The MDA level was quantified using the TBA method (Jiancheng Bioengineering, Nanjing, China), while the GSH level was assessed via the microplate method. Single-cell suspensions of cardiac tissue were prepared through enzymatic digestion, after which the level of ROS was detected using chemical fluorescence (Beyotime, Shanghai, China).

### 4.7. MMP Measurement in the Cardiac Tissues

The MMP was analyzed in accordance with the manufacturer’s instructions (Beyotime, Shanghai, China). Briefly, following heat stress treatment, the cardiac tissue was minced and subjected to low-temperature pancreatic enzyme digestion to isolate the mitochondria. The isolated mitochondria were then incubated with JC-1 staining solution before undergoing time-resolved fluorescence scanning using a fluorescence spectrophotometer, with data recorded throughout the process.

### 4.8. Detection of Cell Apoptosis in the Cardiac Tissues and PCWs

According to the manufacturer’s instructions, the TUNEL cell apoptosis detection kit (Beyotime, Beijing, China) was utilized to assess the level of apoptosis in the cardiac tissue of WCC. Additionally, Hoechst 33342 (Beyotime, Beijing, China) was employed to evaluate the apoptosis level in PCWs. Images were subsequently captured using a fluorescence microscope (NIKON, Ti2-E, Tokyo, Japan).

### 4.9. GA Treatment in PCWs

As a functional inhibitor of HSP90, 0.1 µM GA (Aladdin, Shanghai, China) was administered to the PCWs for 14 h to inhibit the molecular chaperone activity of HSP90. Subsequently, both treated and untreated PCWs were exposed to an incubator preheated to 42 ± 1 °C for 5 h.

### 4.10. Plasmid Construction and Transfection in PCWs

The chicken HSP90 coding sequence (CDS) region (NM_001109785) was sourced from the National Center for Biotechnology Information (NCBI) and synthesized by Wuhan Gene Creation Biological Co., Ltd., Wuhan, China. Subsequently, the synthesized products were cloned into pc3.1 DNA vectors. The recombinant expression plasmids were then sequenced. PCWs were seeded in 6-well plates. The transfection system was prepared following the instructions provided by Lipofectamine 2000 (Invitrogen, Waltham, MA, USA). Specifically, 2.5 μg of plasmid was added to 125 μL of DMEM, mixed gently, and 4 μL of Lipofectamine 2000 was incorporated. The mixture was incubated for 10 min before being added dropwise to the wells. After 6 h, the cell culture medium was replaced with DMEM containing 10% FBS.

### 4.11. Measurement of MDA, GSH and ROS in PCWs

The MDA level was quantified using the TBA method (Jiancheng Bioengineering, Nanjing, China), while the GSH level was measured via the microplate method in PCWs subjected to heat stress, GA treatment, and transfection with an HSP90 overexpression plasmid. Following the washing of cells exposed to heat stress, GA treatment, or transfection with the HSP90 overexpression plasmid using PBS, ROS levels were assessed through chemical fluorescence (Beyotime, Shanghai, China), with all procedures conducted in accordance with the manufacturers’ instructions.

### 4.12. MMP Measurement in PCWs

The MMP was assessed using a mitochondrial membrane potential assay kit (Beyotime, Beijing, China). Briefly, cells were incubated with the working solution of JC-1 at 37 °C for 25 min. Following three washes with PBS, MMP was measured using an F-4700 fluorescence spectrophotometer (HITACHI, Tokyo, Japan). The red-to-green fluorescence ratio was determined to evaluate the MMP.

### 4.13. Immunofluorescence

PCWs transfected with the pc3.1DNA-HSP90 plasmid or subjected to heat stress or GA treatment were placed on glass slides and treated with 100 nM Mit Tracker Deep Red FM (Beyotime, Beijing, China) for 25 min. The cells were then fixed with precooled methanol for 15 min and subsequently blocked with 5% BSA for 1 h. Following this, the cells were incubated overnight at 4 °C with anti-LC3 (1∶500, Proteintech, Wuhan, China), anti-HSP90 (1∶200, Abcam, Cambridge, MA, USA), or anti-Beclin-1 (1:50, Santa Cruz Biotechnology, Santa Cruz, CA, USA) in 1% BSA. After washing, the cells were incubated with FITC-labeled goat anti-rabbit IgG or Cy3-labeled goat anti-mouse IgG (Beyotime, Shanghai, China). Nuclei were stained with DAPI for 10 min. The fluorescent secondary antibodies and DAPI were obtained from Beyotime, Shanghai, China. Cell fluorescence images were captured using an inverted confocal microscope (Axio-Imager LSM-800, Oberkochen, Germany).

### 4.14. Coimmunoprecipitation

Total cardiac protein lysates were prepared, and immunoprecipitation was conducted as previously described [67]. Equal amounts of protein were incubated overnight at 4 °C with anti-HSP90 (1:200). The immunoprecipitated pellets were subsequently incubated with protein A/G agarose beads and subjected to five washes with PBS. The precipitated proteins were collected and separated using 10% SDS-PAGE, with specific proteins detected via Western blotting using the corresponding antibodies. The antibodies were sourced from the following suppliers: Beclin-1 (1:1000, Santa Cruz Biotechnology, Santa Cruz, CA, USA) and anti-HSP90 (1:1000, Proteintech, Wuhan, China).

### 4.15. Western Blotting

Cardiac tissue or PCWs transfected with the pc3.1 DNA-HSP90 plasmid or subjected to heat stress or GA treatment were incubated for 30 min in precooled RIPA lysis buffer supplemented with PMSF (Solarbio, Beijing, China). Protein samples ranging from 10 to 30 µg were separated using 10% to 12% SDS-polyacrylamide gel electrophoresis and subsequently transferred to PVDF membranes. Following a blocking step, the membranes were incubated with diluted primary antibodies for 12 h at 4 °C. After washing the membranes three times with TBST, they were incubated with diluted HRP-conjugated goat anti-rabbit IgG (1:10,000, Abbkine, Beijing, China) or HRP-conjugated goat anti-mouse IgG (1:10,000, Abbkine, Beijing, China) at room temperature for 1 h. The reaction was detected using a hypersensitive ECL chemiluminescence kit (Boster, Wuhan, China). The blots were scanned with an Amersham Imager 600 (GE Healthcare Bio-Sciences AB, Inc., MA, USA), and the gray values were analyzed using ImageJ. The primary antibodies utilized in this study are listed in Table S1.

### 4.16. Statistical Analysis

Statistical analysis was conducted using Prism 8 (GraphPad, La Jolla, CA, USA). The quantitative data are presented as the mean ± SEM. Differences between two groups were evaluated using *t*-tests. while one-way ANOVA with Duncan’s multiple comparisons was employed for comparisons among multiple groups. All *p* values were considered to indicate statistical significance at *p* < 0.05.

## 5. Conclusions

In summary, our study demonstrates that heat stress induces mitochondrial dysfunction and oxidative stress in the cardiomyocytes of WCCs, ultimately leading to apoptosis. Furthermore, heat stress increases the levels of Pink1/Parkin-mediated mitophagy and HSP90 in both WCC cardiomyocytes and PCWs. Furthermore, HSP90 enhances Pink–Parkin-mediated mitophagy by increasing the interaction between HSP90 and Beclin-1, thereby inhibiting oxidative stress and apoptosis in PCWs. Consequently, we have elucidated a novel role for HSP90 and mitophagy in the regulation of acute cardiomyocyte injury induced by heat stress.

## Data Availability

Data will be made available on request.

## Supplementary Materials

The following supporting information can be downloaded at: https://www.mdpi.com/article/10.3390/ijms252111695/s1.

## References

[1] Lv L., van de Pol M., Osmond H.L., Liu Y., Cockburn A., Kruuk L. (2023). Winter mortality of a passerine bird increases following hotter summers and during winters with higher maximum temperatures. Sci. Adv..

[2] Hu R., He Y., Arowolo M.A., Wu S., He J. (2019). Polyphenols as Potential Attenuators of Heat Stress in Poultry Production. Antioxidants.

[3] Nawaz A.H., Lin S., Wang F., Zheng J., Sun J., Zhang W., Jiao Z., Zhu Z., An L., Zhang L. (2023). Investigating the heat tolerance and production performance in local chicken breed having normal and dwarf size. Animal.

[4] Vandana G.D., Sejian V., Lees A.M., Pragna P., Silpa M.V., Maloney S.K. (2021). Heat stress and poultry production: Impact and amelioration. Int. J. Biometeorol..

[5] Zhang X.H., Wu J.X., Sha J.Z., Yang B., Sun J.R., Bao E.D. (2020). Heat shock protein 90 relieves heat stress damage of myocardial cells by regulating Akt and PKM2 signaling in vivo. Int. J. Mol. Med..

[6] Wu D., Zhang M., Lu Y., Tang S., Kemper N., Hartung J., Bao E. (2016). Aspirin-induced heat stress resistance in chicken myocardial cells can be suppressed by BAPTA-AM in vitro. Cell Stress Chaperones.

[7] Yin B., Di L., Tang S., Bao E. (2020). Vitamin CNa enhances the antioxidant ability of chicken myocardium cells and induces heat shock proteins to relieve heat stress injury. Res. Vet. Sci..

[8] Bou-Teen D., Kaludercic N., Weissman D., Turan B., Maack C., Di Lisa F., Ruiz-Meana M. (2021). Mitochondrial ROS and mitochondria-targeted antioxidants in the aged heart. Free. Radic. Biol. Med..

[9] Saito T., Hamano K., Sadoshima J. (2021). Molecular mechanisms and clinical implications of multiple forms of mitophagy in the heart. Cardiovasc. Res..

[10] Ryter S.W., Choi A.M. (2013). Autophagy: An Integral Component of the Mammalian Stress Response. J. Biochem. Pharmacol. Res..

[11] Titus A.S., Sung E.A., Zablocki D., Sadoshima J. (2023). Mitophagy for cardioprotection. Basic. Res. Cardiol..

[12] Shoji-Kawata S., Sumpter R., Leveno M., Campbell G.R., Zou Z., Kinch L., Wilkins A.D., Sun Q., Pallauf K., MacDuff D. (2013). Identification of a candidate therapeutic autophagy-inducing peptide. Nature.

[13] Peugnet V., Chwastyniak M., Mulder P., Lancel S., Bultot L., Fourny N., Renguet E., Bugger H., Beseme O., Loyens A. (2022). Mitochondrial-Targeted Therapies Require Mitophagy to Prevent Oxidative Stress Induced by SOD2 Inactivation in Hypertrophied Cardiomyocytes. Antioxidants.

[14] Chen Y., Culetto E., Legouis R. (2021). A DRP-1 dependent autophagy process facilitates rebuilding of the mitochondrial network and modulates adaptation capacity in response to acute heat stress during C. elegans development. Autophagy.

[15] Yang C., Luo P., Yang Y.T., Fu X.L., Li B.X., Shen X., Xu D.N., Huang Y.M., Tian Y.B., Liu W.J. (2024). Drp1 regulated PINK1-dependent mitophagy protected duck follicular granulosa cells from acute heat stress injury. Poult. Sci..

[16] Xie J., Tang L., Lu L., Zhang L., Xi L., Liu H.C., Odle J., Luo X. (2014). Differential expression of heat shock transcription factors and heat shock proteins after acute and chronic heat stress in laying chickens (*Gallus gallus*). PLoS ONE.

[17] Dabravolski S.A., Sukhorukov V.N., Kalmykov V.A., Orekhov N.A., Grechko A.V., Orekhov A.N. (2022). Heat Shock Protein 90 as Therapeutic Target for CVDs and Heart Ageing. Int. J. Mol. Sci..

[18] Quel N.G., Pinheiro G., Rodrigues L., Barbosa L., Houry W.A., Ramos C. (2020). Heat shock protein 90 kDa (Hsp90) from Aedes aegypti has an open conformation and is expressed under heat stress. Int. J. Biol. Macromol..

[19] Zhang X.H., Zhu H.S., Qian Z., Tang S., Wu D., Kemper N., Hartung J., Bao E.D. (2016). The association of Hsp90 expression induced by aspirin with anti-stress damage in chicken myocardial cells. J. Vet. Sci..

[20] Yao X., Zhu J., Li L., Yang B., Chen B., Bao E., Zhang X. (2023). Hsp90 protected chicken primary myocardial cells from heat-stress injury by inhibiting oxidative stress and calcium overload in mitochondria. Biochem. Pharmacol..

[21] Tian S., Tang W., Zhong Z., Wang Z., Xie X., Liu H., Chen F., Liu J., Han Y., Qin Y. (2023). Identification of Runs of Homozygosity Islands and Functional Variants in Wenchang Chicken. Animal.

[22] Cui M., Wang Z., Chen K., Shah A.M., Tan W., Duan L., Sanchez-Ortiz E., Li H., Xu L., Liu N. (2020). Dynamic Transcriptional Responses to Injury of Regenerative and Non-regenerative Cardiomyocytes Revealed by Single-Nucleus RNA Sequencing. Dev. Cell.

[23] Marie M., Bigot K., Angebault C., Barrau C., Gondouin P., Pagan D., Fouquet S., Villette T., Sahel J.A., Lenaers G. (2018). Light action spectrum on oxidative stress and mitochondrial damage in A2E-loaded retinal pigment epithelium cells. Cell Death Dis..

[24] Zhu X., Zou W., Meng X., Ji J., Wang X., Shu H., Chen Y., Pan D., Wang K., Zhou F. (2022). Elaiophylin Inhibits Tumorigenesis of Human Uveal Melanoma by Suppressing Mitophagy and Inducing Oxidative Stress via Modulating SIRT1/FoxO3a Signaling. Front. Oncol..

[25] Xu C., Liu J., Hsu L.C., Luo Y., Xiang R., Chuang T.H. (2011). Functional interaction of heat shock protein 90 and Beclin 1 modulates Toll-like receptor-mediated autophagy. FASEB J..

[26] Kikusato M., Xue G., Pastor A., Niewold T.A., Toyomizu M. (2021). Effects of plant-derived isoquinoline alkaloids on growth performance and intestinal function of broiler chickens under heat stress. Poult. Sci..

[27] Tang S., Yin B., Xu J., Bao E. (2018). Rosemary Reduces Heat Stress by Inducing CRYAB and HSP70 Expression in Broiler Chickens. Oxidative Med. Cell. Longev..

[28] Guo T., Chen M., Liu J., Wei Z., Yuan J., Wu W., Wu Z., Lai Y., Zhao Z., Chen H. (2023). Neuropilin-1 promotes mitochondrial structural repair and functional recovery in rats with cerebral ischemia. J. Transl. Med..

[29] Hu H., Bai X., Xu K., Zhang C., Chen L. (2021). Effect of phloretin on growth performance, serum biochemical parameters and antioxidant profile in heat-stressed broilers. Poult. Sci..

[30] Song E., Tang S., Xu J., Yin B., Bao E., Hartung J. (2016). Lenti-siRNA Hsp60 promote bax in mitochondria and induces apoptosis during heat stress. Biochem. Biophys. Res. Commun..

[31] Chen Z., Huang L., Tso A., Wang S., Fang X., Ouyang K., Han Z. (2021). Mitochondrial Chaperones and Proteases in Cardiomyocytes and Heart Failure. Front. Mol. Biosci..

[32] Yu Z., Hou Y., Zhou W., Zhao Z., Liu Z., Fu A. (2021). The effect of mitochondrial transplantation therapy from different gender on inhibiting cell proliferation of malignant melanoma. Int. J. Biol. Sci..

[33] Xu D., Li B., Cao N., Li W., Tian Y., Huang Y. (2017). The protective effects of polysaccharide of Atractylodes macrocephala Koidz (PAMK) on the chicken spleen under heat stress via antagonizing apoptosis and restoring the immune function. Oncotarget.

[34] Qian L., Song X., Ren H., Gong J., Cheng S. (2004). Mitochondrial mechanism of heat stress-induced injury in rat cardiomyocyte. Cell Stress Chaperones.

[35] Yang Y., Xing D., Zhou F., Chen Q. (2010). Mitochondrial autophagy protects against heat shock-induced apoptosis through reducing cytosolic cytochrome c release and downstream caspase-3 activation. Biochem. Biophys. Res. Commun..

[36] Song P., Sun M., Liu C., Liu J., Lin P., Chen H., Zhou D., Tang K., Wang A., Jin Y. (2023). Reactive Oxygen Species Damage Bovine Endometrial Epithelial Cells via the Cytochrome C-mPTP Pathway. Antioxidants.

[37] Jin S.M., Youle R.J. (2013). The accumulation of misfolded proteins in the mitochondrial matrix is sensed by PINK1 to induce PARK2/Parkin-mediated mitophagy of polarized mitochondria. Autophagy.

[38] Uoselis L., Nguyen T.N., Lazarou M. (2023). Mitochondrial degradation: Mitophagy and beyond. Mol. Cell.

[39] Allen G.F., Toth R., James J., Ganley I.G. (2013). Loss of iron triggers PINK1/Parkin-independent mitophagy. EMBO Rep..

[40] Gladkova C., Maslen S., Skehel J.M., Komander D. (2018). Mechanism of parkin activation by PINK1. Nature.

[41] Choi S.Y., Park J.S., Shon C.H., Lee C.Y., Ryu J.M., Son D.J., Hwang B.Y., Yoo H.S., Cho Y.C., Lee J. (2019). Fermented Korean Red Ginseng Extract Enriched in Rd and Rg3 Protects against Non-Alcoholic Fatty Liver Disease through Regulation of mTORC1. Nutrure.

[42] Carreira R.S., Lee Y., Ghochani M., Gustafsson A.B., Gottlieb R.A. (2010). Cyclophilin D is required for mitochondrial removal by autophagy in cardiac cells. Autophagy.

[43] Chemaly E.R., Hadri L., Zhang S., Kim M., Kohlbrenner E., Sheng J., Liang L., Chen J., K-Raman P., Hajjar R.J. (2011). Long-term in vivo resistin overexpression induces myocardial dysfunction and remodeling in rats. J. Mol. Cell. Cardiol..

[44] Yang X., Wang N., Ren S., Hu Y., Wang H., Ji A., Cao L.H., Li M., Liu J., Wang H. (2022). Phosphorylation regulation of cardiac proteins in Babesia microti infected mice in an effort to restore heart function. Parasites Vectors.

[45] Meng T., Deng J., Xiao D., Arowolo M.A., Liu C., Chen L., Deng W., He S., He J. (2022). Protective Effects and Potential Mechanisms of Dietary Resveratrol Supplementation on the Spleen of Broilers Under Heat Stress. Front. Nutr..

[46] Wang Y., Yang C., Elsheikh N.A.H., Li C., Yang F., Wang G., Li L. (2019). HO-1 reduces heat stress-induced apoptosis in bovine granulosa cells by suppressing oxidative stress. Aging.

[47] Faridi U., Dhawan S.S., Pal S., Gupta S., Shukla A.K., Darokar M.P., Sharma A., Shasany A.K. (2016). Repurposing L-Menthol for Systems Medicine and Cancer Therapeutics? L-Menthol Induces Apoptosis through Caspase 10 and by Suppressing HSP90. OMICS.

[48] Lu Q., Sakhatskyy P., Newton J., Shamirian P., Hsiao V., Curren S., Gabino M.G., Pedroza M., Blackburn M.R., Rounds S. (2013). Sustained adenosine exposure causes lung endothelial apoptosis: A possible contributor to cigarette smoke-induced endothelial apoptosis and lung injury. Am. J. Physiol.-Lung Cell. Mol. Physiol..

[49] Li B., Lin Q., Guo H., Liu L., Li Y. (2019). Ghrelin regulates sepsis-induced rat acute gastric injury. Mol. Med. Rep..

[50] Li M., Hassan F.U., Tang Z., Peng L., Liang X., Li L., Peng K., Xie F., Yang C. (2020). Mulberry Leaf Flavonoids Improve Milk Production, Antioxidant, and Metabolic Status of Water Buffaloes. Front. Vet. Sci..

[51] Xu J., Tang S., Yin B., Sun J., Song E., Bao E. (2019). Correction to: Co-enzyme Q10 and acetyl salicylic acid enhance Hsp70 expression in primary chicken myocardial cells to protect the cells during heat stress. Mol. Cell. Biochem..

[52] Tsutsumi S., Mollapour M., Prodromou C., Lee C.T., Panaretou B., Yoshida S., Mayer M., Neckers L. (2012). Charged linker sequence modulates eukaryotic heat shock protein 90 (Hsp90) chaperone activity. Proc. Natl. Acad. Sci. USA.

[53] Wang Y., Xu E., Musich P.R., Lin F. (2019). Mitochondrial dysfunction in neurodegenerative diseases and the potential countermeasure. CNS Neurosci. Ther..

[54] Semanchik P.L., Wesolowski L.T., Simons J.L., Freestone A., Rudolph T.E., Roths M., Rhoads R.P., Baumgard L.H., Selsby J.T., Springer S.H.W. (2022). Heat Stress More Negatively Impacts Cardiac Muscle Mitochondria in Female Versus Male Pigs. FASEB J..

[55] Zitzmann K., Ailer G., Vlotides G., Spoettl G., Maurer J., Göke B., Beuschlein F., Auernhammer C.J. (2013). Potent antitumor activity of the novel HSP90 inhibitors AUY922 and HSP990 in neuroendocrine carcinoid cells. Int. J. Oncol..

[56] Islam A., Rehana B., Zhang M., Liu Z.J., Tang S., Hartung J., Bao E.D. (2014). Expression of heat shock protein 90 alpha (Hsp90alpha) in primary neonatal rat myocardial cells exposed to various periods of heat stress in vitro. Genet. Mol. Res..

[57] Hu L., Fang H., Abbas Z., Luo H., Brito L.F., Wang Y., Xu Q. (2024). The HSP90AA1 gene is involved in heat stress responses and its functional genetic polymorphisms are associated with heat tolerance in Holstein cows. J. Dairy Sci..

[58] Chen B., Yang B., Zhu J., Wu J., Sha J., Sun J., Bao E., Zhang X. (2020). Hsp90 Relieves Heat Stress-Induced Damage in Mouse Kidneys: Involvement of Antiapoptotic PKM2-AKT and Autophagic HIF-1α Signaling. Int. J. Mol. Sci..

[59] Hu B., Zhang Y., Jia L., Wu H., Fan C., Sun Y., Ye C., Liao M., Zhou J. (2015). Binding of the pathogen receptor HSP90AA1 to avibirnavirus VP2 induces autophagy by inactivating the AKT-MTOR pathway. Autophagy.

[60] He W., Ye X., Huang X., Lel W., You L., Wang L., Chen X., Qian W. (2016). Hsp90 inhibitor, BIIB021, induces apoptosis and autophagy by regulating mTOR-Ulk1 pathway in imatinib-sensitive and -resistant chronic myeloid leukemia cells. Int. J. Oncol..

[61] Kimura T., Uesugi M., Takase K., Miyamoto N., Sawada K. (2017). Hsp90 inhibitor geldanamycin attenuates the cytotoxicity of sunitinib in cardiomyocytes via inhibition of the autophagy pathway. Toxicol. Appl. Pharmacol..

[62] Joo J.H., Dorsey F.C., Joshi A., Hennessy-Walters K.M., Rose K.L., McCastlain K., Zhang J., Iyengar R., Jung C.H., Suen D.F. (2011). Hsp90-Cdc37 chaperone complex regulates Ulk1- and Atg13-mediated mitophagy. Mol. Cell..

[63] Li L., Cui Y.J., Liu Y., Li H.X., Su Y.D., Li S.N., Wang L.L., Zhao Y.W., Wang S.X., Yan F. (2022). ATP6AP2 knockdown in cardiomyocyte deteriorates heart function via compromising autophagic flux and NLRP3 inflammasome activation. Cell Death Discov..

[64] Quiles J.M., Najor R.H., Gonzalez E., Jeung M., Liang W., Burbach S.M., Zumaya E.A., Diao R.Y., Lampert M.A., Gustafsson A.B. (2023). Deciphering functional roles and interplay between Beclin1 and Beclin2 in autophagosome formation and mitophagy. Sci. Signal..

[65] Hasan A., Haque E., Hameed R., Maier P.N., Irfan S., Kamil M., Nazir A., Mir S.S. (2020). Hsp90 inhibitor gedunin causes apoptosis in A549 lung cancer cells by disrupting Hsp90: Beclin-1:Bcl-2 interaction and downregulating autophagy. Life Sci..

[66] Jamal Z., Das J., Ghosh S., Gupta A., Chattopadhyay S., Chatterji U. (2020). Arsenic-induced immunomodulatory effects disorient the survival-death interface by stabilizing the Hsp90/Beclin1 interaction. Chemosphere.

[67] Zhong F.Y., Zhao Y.C., Zhao C.X., Gu Z.C., Lu X.Y., Jiang W.L., Gao L.C., Li W.L., Qin Z.H., Ge H. (2022). The Role of CD147 in Pathological Cardiac Hypertrophy Is Regulated by Glycosylation. Oxidative Med. Cell. Longev..

