# Supplementary material for: HSP90 Enhances Mitophagy to Improve the Resistance of Car-Diomyocytes to Heat Stress in Wenchang Chickens
Source: Int J Mol Sci. 2024 Oct 30;25(21):11695. doi: 10.3390/ijms252111695 (PMC11546521; doi:10.3390/ijms252111695)
Supplement: Supplementary file 1 [file ijms-25-11695-s001.zip › ijms-3254879-supplementary.pdf]

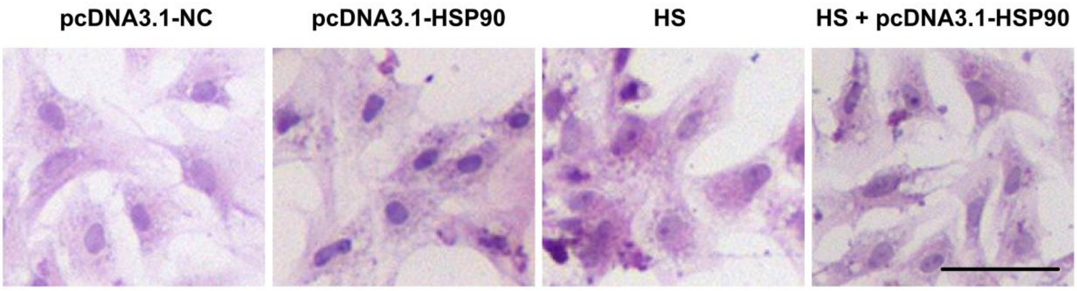

Figure S1. Representative image of H&E staining of PCWs with or without HSP90 overexpression after heat stress (Scale bars, 20 μm)

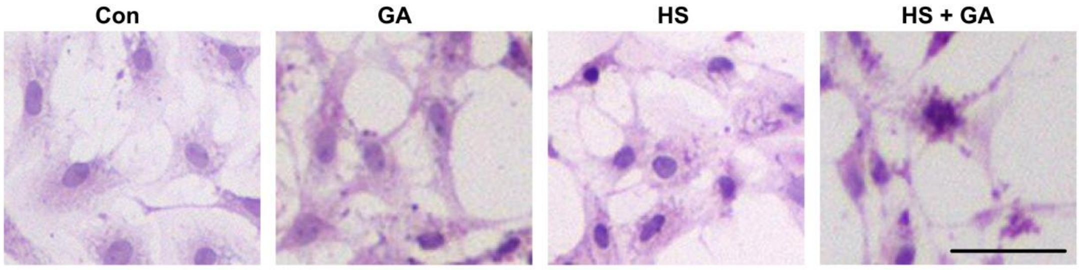

Figure S2. Representative image of H&E staining of PCWs treated with or without GA after heat stress (Scale bars, 20 μm)

Table S1. Primary antibodies used for immunofluorescence (IF) and western blot (WB) analysis in the study

Antibody	Source	Identifier	Dilution
Pink1	Santa Cruz	Cat# sc-517353	1:1000
Parkin	Santa Cruz	Cat# sc-32282	1:1000
Beclin-1	Santa Cruz	Cat# sc-48341	1:1000
P62	Novus Biologicals	Cat# H00008878-M01	1:2000
Bcl-2	Wanleibio	Cat# WL01556	1:1000
Bax	Santa Cruz	Cat# sc-65532	1:1000
Caspase3	Wanleibio	Cat# WL02117	1:1000
Cleaved caspase3	Cell Signaling	Cat# 9661	1:1000
LC3	Cell Signaling	Cat# 4108S	1:1000
HSP90	Proteintech	Cat# 13171-1-AP	1:1000
GAPDH	Abbkine	Cat# ABL1020	1:10000
